# Baseline Monitoring of the Western Arctic Ocean Estimates 20% of Canadian Basin Surface Waters Are Undersaturated with Respect to Aragonite

**DOI:** 10.1371/journal.pone.0073796

**Published:** 2013-09-11

**Authors:** Lisa L. Robbins, Jonathan G. Wynn, John T. Lisle, Kimberly K. Yates, Paul O. Knorr, Robert H. Byrne, Xuewu Liu, Mark C. Patsavas, Kumiko Azetsu-Scott, Taro Takahashi

**Affiliations:** 1 St. Petersburg Coastal and Marine Science Center, United States Geological Survey, St. Petersburg, Florida, United States of America; 2 Department of Geology, University of South Florida, Tampa, Florida, United States of America; 3 College of Marine Science, University of South Florida, St. Petersburg, Florida, United States of America; 4 Ocean Sciences Division, Department of Fisheries and Oceans, Bedford Institute of Oceanography, Dartmouth, Nova Scotia, Canada; 5 Lamont-Doherty Earth Observatory of Columbia University, Palisades, New York, United States of America; Instituto de Biologia, Brazil

## Abstract

Marine surface waters are being acidified due to uptake of anthropogenic carbon dioxide, resulting in surface ocean areas of undersaturation with respect to carbonate minerals, including aragonite. In the Arctic Ocean, acidification is expected to occur at an accelerated rate with respect to the global oceans, but a paucity of baseline data has limited our understanding of the extent of Arctic undersaturation and of regional variations in rates and causes. The lack of data has also hindered refinement of models aimed at projecting future trends of ocean acidification. Here, based on more than 34,000 data records collected in 2010 and 2011, we establish a baseline of inorganic carbon data (*p*H, total alkalinity, dissolved inorganic carbon, partial pressure of carbon dioxide, and aragonite saturation index) for the western Arctic Ocean. This data set documents aragonite undersaturation in ∼20% of the surface waters of the combined Canada and Makarov basins, an area characterized by recent acceleration of sea ice loss. Conservative tracer studies using stable oxygen isotopic data from 307 sites show that while the entire surface of this area receives abundant freshwater from meteoric sources, freshwater from sea ice melt is most closely linked to the areas of carbonate mineral undersaturation. These data link the Arctic Ocean’s largest area of aragonite undersaturation to sea ice melt and atmospheric CO_2_ absorption in areas of low buffering capacity. Some relatively supersaturated areas can be linked to localized biological activity. Collectively, these observations can be used to project trends of ocean acidification in higher latitude marine surface waters where inorganic carbon chemistry is largely influenced by sea ice meltwater.

## Introduction

The importance of the Arctic Ocean in the context of global carbon dioxide (CO_2_) uptake and ocean acidification is widely accepted [1]–[9]. Ocean acidification is projected to occur relatively rapidly in the Arctic due to processes and conditions that are unique to Arctic surface waters [7]–[9]. For instance, a large fraction of the global net CO_2_ uptake during recent decades (*∼*2200 Tg C yr^−1^) has occurred over the relatively small surface area of the Arctic Ocean. From the estimated net CO_2_ uptake rates of the Arctic Ocean (65–175 Tg C yr^−1^; reviewed in ref. [8]), it is inferred that as much as 7.5% of global oceanic CO_2_ uptake may occur in the Arctic Ocean, which comprises only 3.9% of the global ocean’s surface. These figures may be even more noteworthy given that the uptake occurs predominantly in seasonally ice-free areas, which are a fraction of the Arctic Ocean surface.

One driving factor behind the disproportionate Arctic Ocean CO_2_ uptake and resulting acidification is the relatively cold surface water, which absorbs more CO_2_ than warmer seawater [3], [5]. Furthermore, over the past decade, summer sea ice extent has rapidly declined [9]–[13], reaching a record low in 2012 [14]. Multiyear sea ice has experienced the greatest decline [9]. This loss of multiyear ice has exposed the surface mixed layer (typically≤50 m thick), which is undersaturated with respect to atmospheric CO_2_; the result is oceanic CO_2_ uptake. Previously, ice cover significantly inhibited CO_2_ exchange between this layer and the atmosphere [15]–[17].

This melt-associated exposure of undersaturated waters is unique to the Arctic, where a steep vertical density gradient, attributable primarily to a cold halocline underlying the surface mixed layer, inhibits upward mixing of CO_2_-rich deep waters, even during winter when the mixed layer temperature is lowest and salinity is highest due to sea ice formation [18]. Unlike the Southern Ocean seasonal ice zone, where the under-ice water has a relatively high partial pressure of CO_2_ (*p*CO_2_∼420 µatm) due to rapid vertical mixing of Circumpolar Deep Water [19], the mixed layer immediately under Arctic sea ice remains low in salinity and *p*CO_2_. Thus, when Arctic sea ice melts in summer, the under-ice layer is exposed to the atmosphere and is also diluted by freshwater from sea ice melt. This dilution is an important consequence of multiyear sea ice loss because it reduces surface seawater calcium and carbonate ion concentrations and *p*H [20], [21]. The combination of these processes is expected to drive the expansion of areas of carbonate mineral undersaturation in the Arctic Ocean over the next decades [3]–[6].

The in situ melting of sea ice has a number of direct consequences for seawater chemistry. The saturation state index of carbonate minerals (Ω_carb_) is defined by:
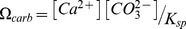
where [Ca^2+^] is the calcium ion concentration, [CO_3_^2–^] is the carbonate ion concentration, and *K*_sp_ is the stoichiometric solubility product of the mineral. This index provides a quantitative measure of the thermodynamic potential for carbonate minerals such as aragonite and calcite to either precipitate (Ω_carb_>1) or dissolve (Ω_carb_<1). Importantly, when multiyear sea ice melts, fresh water containing relatively little carbonate and calcium is liberated, thereby lowering Ω_carb_ of the receiving seawater. The resulting changes in *p*H, Ω_carb_, and *p*CO_2_ affect calcifying organisms at the base of the food chain, creating effects that propagate through higher trophic levels [1], [3]. Thus, the expansion of acidification into areas previously isolated from atmospheric contact by multiyear ice cover is likely to have ecological and economic consequences.

Ocean acidification models project that under a number of plausible scenarios of increasing atmospheric CO_2_, the Arctic Ocean will become undersaturated with respect to carbonate minerals in the next decade [3]–[6]. Recent data indicate that some areas are already undersaturated with respect to the carbonate mineral aragonite [16], [21]–[25]. These areas, which include regions of the Canadian Archipelago, Canada Basin, and Beaufort Sea, exhibit the lowest aragonite saturation state (Ω_a_) values during late summer, when sea ice is at its minimum annual extent. However, the spatial extent of undersaturated waters in the western Arctic Ocean has been poorly constrained.

**Figure 1:**
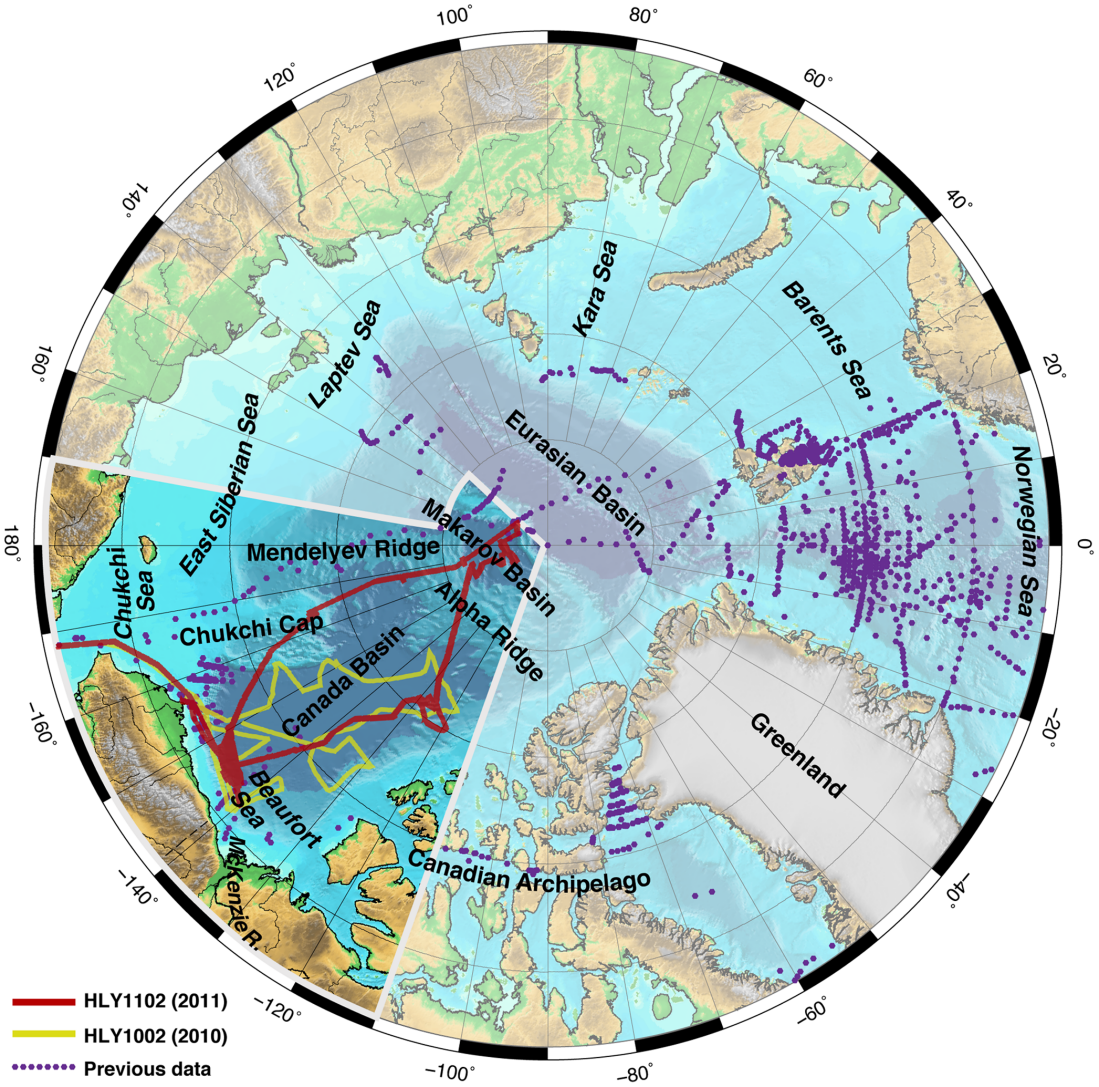
Location map of Arctic Ocean underway and discrete inorganic carbon data. Locations are shown for the HLY1002 and HLY1102 cruises, as well as previous work. Previous data are from the Carbon Dioxide Information Analysis Center (http://cdiac.ornl.gov/oceans/datmet.html), Japan Agency for Marine Earth Science and Technology (http://www.godac.jamstec.go.jp/dataportal/viewer.htm), and BioChem (http://www.meds-sdmm.dfo-mpo.gc.ca/BioChem/biochem-eng.htm). Digital bathymetry is from the International Bathymetric Chart of the Arctic Ocean [48]. The highlighted sector is shown in subsequent figures.

Much of the uncertainty of models that project future trends of Arctic Ocean acidification is due to inadequate data coverage, particularly in higher latitudes. As of late 2011, only ∼20,000 data points were available in public databases for the entire Arctic Ocean (surface and water column; data from refs. [16], [17], [21]–[26] and the data sets of the Japan Agency for Marine Earth Science and Technology, BioChem, and the Carbon Dioxide Information Analysis Center, including CARINA and GLODAP; Figure 1). Most of the data are from the Atlantic/Norwegian Sea (Figure 1). Establishing carbonate chemistry baselines and improving our understanding of the processes that drive changes in carbonate chemistry are fundamental for constraining the biogeochemical process parameters needed to predict ocean acidification effects on Arctic Ocean flora and fauna. Thus, a systematic approach for measuring the carbonate chemistry of the Arctic Ocean at high spatial resolution from coastal to pelagic waters is needed.

To begin to establish these baselines and fill critical data gaps, we collected carbonate chemistry data at high spatial resolution in the western Arctic Ocean during 2010 and 2011. We supplemented these data with measurements of conservative tracers of water sources as well as proxies of biological processes. This study compares aragonite saturation states in open pelagic waters, shallow shelf waters, and ice-bound high-latitude waters to delineate rates of change and causes of variation in carbonate mineral saturation states.

## Materials and Methods

From 3 August to 5 September 2010 and from 16 August to 28 September 2011, we collected seawater chemical and biological measurements as noninterference science on United States (U.S.) Extended Continental Shelf Task Force cruises aboard the U.S. Coast Guard Cutter *Healy* in the western Arctic Ocean (Figure 1). No specific permissions were required for collecting water samples in Arctic international waters or U.S. or Canadian waters. Our studies did not involve endangered or protected species. Continuous and discrete water samples (Table 1) were collected underway from ∼8 m below the waterline, via a port in the ship’s hull. Approximately 25,000 data records resulted from the 2010 cruise; approximately 9,000 data records resulted from the 2011 cruise.

**Table 1:** Methods used to measure carbonate system parameters on the HLY1002 (2010) and HLY1102 (2011) cruises.

Sample Type	Analysis	Parameter	Approx. sample interval (year)
Continuous (flow-through)	USF Multiparameter Inorganic CarbonAnalyzer (MICA) – spectrophotometric –on ship	*p*H, *p*CO_2,_ *T*CO_2_	2 min (2010); 7 min (2011)
Continuous (flow-through)	LDEO Underway system for surfacewater *p*CO_2_– infrared gas analyzer –on ship	*p*CO_2_	3 min (2011)
Discrete (optical cell)	Spectrophotometric – on ship	*p*H	1–4 hr (2010); 1 hr (2011)
Discrete (bottle)	Coulometric, titrimetric – land-based lab	*T*CO_2_, *TA*	8 hr (2010); 1–4 hr (2011)
Discrete (bottle)	Isotope ratio mass spectrometry– land-based lab	*δ^18^O*	8 hr (2010); 1–4 hr (2011)

### Underway Continuous Measurements

Geographic position, salinity (*S*), temperature (*T*), and fluorometric data (*n* = 62,387) were collected using a shipboard Ashtech ADU5 GPS system, a SeaBird SBE45 thermosalinograph, and a Seapoint Chlorophyll Fluorometer [27]. The majority of the carbonate chemical measurements were made using a Multiparameter Inorganic Carbon Analyzer (MICA) [28], a flowthrough system which measured *p*H, *p*CO_2_, and total dissolved inorganic carbon (*T*CO_2_), along with *S* and *T* (*n* = 34,000). Data were logged approximately every 2 mins on the 2010 cruise and every 7 mins on the 2011 cruise. The MICA was calibrated using Certified Reference Material from Professor A. Dickson of the University of California at San Diego. Precision was ±0.002 for *p*H, ±2 µatm for *p*CO_2_, and ±2 µmol kg^−1^ for *T*CO_2_. A Lamont-Doherty Earth Observatory underway system for surface water measurements was also used to measure *p*CO_2_ during the 2011 cruise (*n* = 18,500). This system was calibrated every 4 hrs using five calibration gas mixtures certified by the National Oceanic and Atmospheric Administration (NOAA) Climate Monitoring and Diagnostic Laboratory. The precision of the *p*CO_2_ seawater values is about ±1.5 µatm.

### Discrete Surface Samples and Analyses

Discrete surface water samples were collected from the underway system every 1–4 hrs following the protocols of ref. [29]. The samples (*n*≅560) were immediately analyzed for *p*H using purified meta-cresol purple indicator dye and the method and equations of ref. [30].

Additional discrete samples were collected every 1–8 hrs for post-cruise laboratory analysis of *TA* and *T*CO_2_ (*n*≅305) as well as oxygen isotopic composition (*n*≅307). *TA* was measured spectrophotometrically and *T*CO_2_ was measured coulometrically, both following the procedures of ref. [29], at the Carbon Analysis Laboratory of the U.S. Geological Survey (USGS) St. Petersburg Coastal and Marine Science Center. Stable oxygen isotopic analyses were completed at the University of South Florida Department of Geology’s Stable Isotope Laboratory with a Thermo-Finnigan Delta V 3 keV Isotope Ratio Mass Spectrometer coupled to a Gasbench II preparation device. Stable isotopic data are expressed in the conventional delta (*δ*) notation: *δ^18^O* = [(^18^O/^16^O_sample_−^18^O/^16^O_standard_)/(^18^O/^16^O_standard_)] where *δ* is expressed in per mil (‰) with respect to the VSMOW standard scale. Analytical precision (2σ) on these standards was better than 0.15‰ and 0.10‰ for δ^18^O from HLY1002 and HLY1102, respectively.

### Water Mass Mixing Model

To identify sources of water masses, we applied a three-component mixing model based on measurements of salinity (*S*) and oxygen isotopic composition (*δ^18^O*). This mixing model is well suited to quantify fractional contributions (*f*) from various sources–in this case, meteoric fresh water and sea ice meltwater mixing with seawater. Meteoric freshwater (MW), derived from terrestrial runoff and direct precipitation, has negligible salinity (*S*≅0) but is relatively ^18^O-depleted in the high latitudes of the Arctic (*δ^18^O*≅–20‰). Sea ice melt (SIM) also has relatively low salinity (*S*<5), but its oxygen isotopic composition is similar to that of seawater, from which it is formed (*δ^18^O*≅0 to –6‰). Seawater (SW) has salinity of 32–35 and *δ^18^O* values of –1 to +0.3‰, although these values vary between Atlantic and Pacific source regions. This freshwater tracer method, which takes advantage of the large differences in the *δ^18^O* values of the freshwater sources [31], has been used for decades to partition surface waters into constituent water sources [32]–[38].

Using discrete measurements of *S* and *δ^18^O*, each surface water sample can be partitioned into a mixture of the three end-members, with the fractional contribution of each end-member determined by mass balance:
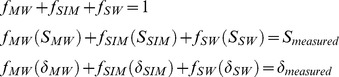
where *δ* is shorthand notation for *δ^18^O*. We parameterize the model with estimates of *S* and *δ^18^O* values (*δ*) of each of the three end-members. For the MW end-member, we use data from recent stream gauging studies, which provide an estimate of the flow-weighted *δ^18^O* value from major Arctic rivers: *S*_MW_ = 0 and *δ^18^O*_MW_ = −20±1 ‰; [34], [35], [39]. Direct precipitation is estimated to have a similar *δ^18^O* value. For the SIM end-member, we use salinity values of multiyear ice: *S*_SIM_ = 4 (ref. [40]). We calculate the *δ^18^O* value of sea ice from the *δ^18^O* value measured in the local surface water plus a fractionation factor for liquid–solid water isotopic fractionation: *δ^18^O*_SIM_ *= δ^18^O*_measured_+2.6‰ [33], [41]. Each cruise measurement of *δ^18^O* therefore provides an individual estimate of *δ^18^O*_SIM_. This approach, which differs somewhat from the static *δ^18^O*_SIM_ approach used in some recent work [35], [38], may be preferable in that a variable *δ^18^O*_SIM_ value may take into account annual changes in the *δ^18^O* values of both seawater and newly formed sea ice. For the SW end-member, we use estimates of the composition of the inflow from the Atlantic (*S*_SW_ = 34.92±0.05 and *δ^18^O*_SW_ = 0.3±0.1 ‰), although we recognize that Arctic seawater, particularly in the southern Canada Basin, is partially derived from Bering Strait Inflow (BSI), which typically has lower salinity and *δ^18^O* values (*S*_BSI_ = 32.5 and *δ^18^O*_BSI_ = −0.8±0.1 ‰; ref. [38]) due to dilution by Bering Sea freshwater inputs. Previous studies have used a variety of characterizations for the seawater end-member in the western Arctic [32], [38], [42]. The advantage of using Atlantic inflow seawater rather than BSI seawater for SW end-member values is that we are then able to make comparisons with other pan-Arctic studies of water sources. The disadvantage is that our calculated *f*_MW_ values may be overestimates. In other words, our calculated *f*_MW_ values, attributed to Arctic river runoff and direct precipitation, may also reflect the influence of freshwater that derives from BSI. Adding dissolved silica measurements to the mass balance model could provide a means of independently quantifying the additional BSI source [34], [38].

**Figure 2:**
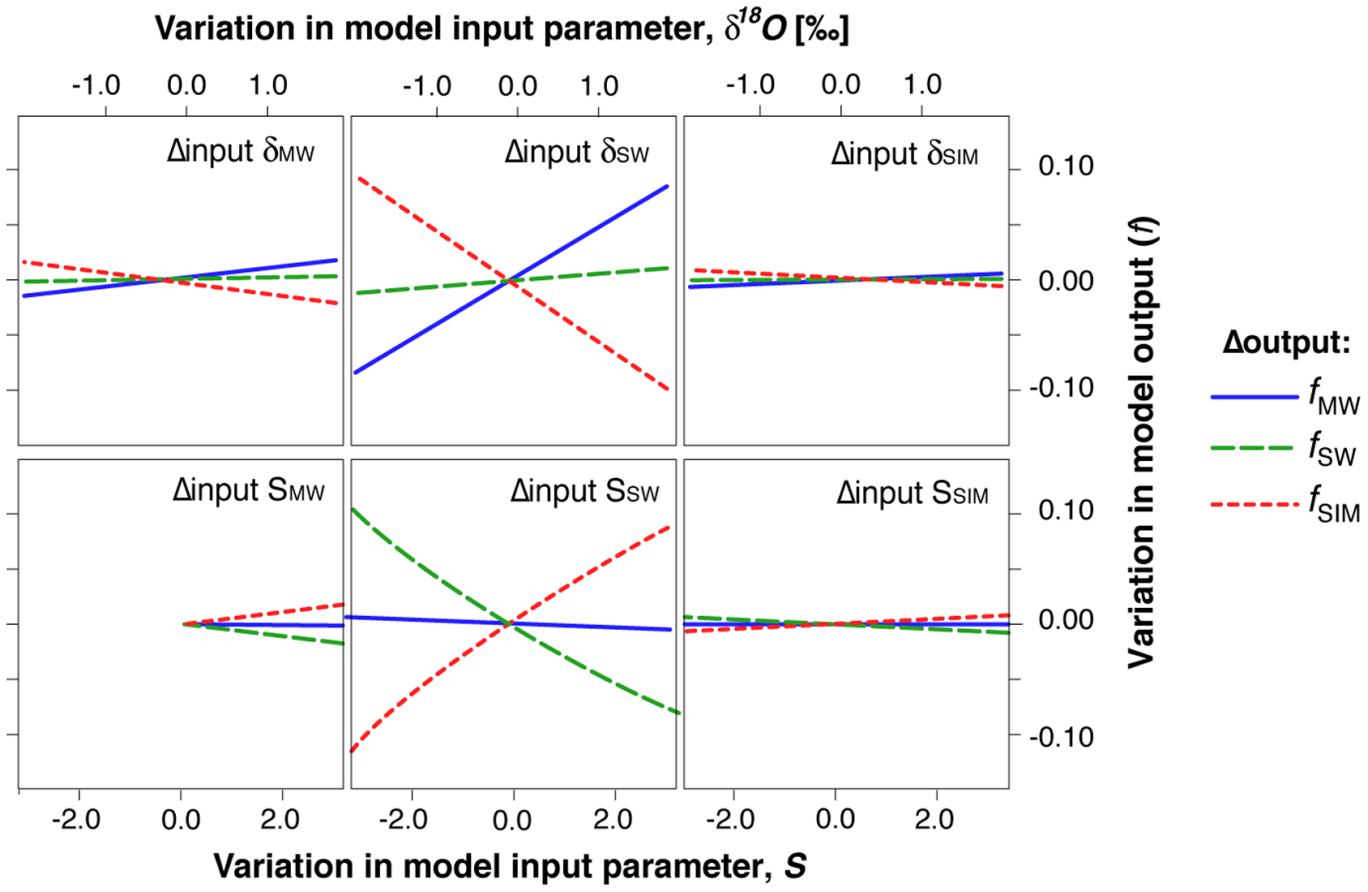
Sensitivity of mixing-model output (*f*_SW_, *f*_SIM_, and *f*_MW_) to variations in assumed end-member compositions. Upper panels show the effect of varying end-member δ^18^O by 10% of the observed δ^18^O range: i.e., by ±0.1(δ^18^O_max_−δ^18^O_min_) = ±0.2‰. Lower panels show the effect of varying end-member *S* by 10% of the observed *S* range: i.e., by ±0.1(*S*_max_−*S*_min_). Model sensitivity is calculated for a hypothetical sample with *S* and δ^18^O equal to the mid-range values of the combined HLY1002 and HLY1102 data sets: *S*_model_ = *S*_min_+½(range S) and δ^18^O_model_ = δ^18^O_min_+½(range δ^18^O).

Figure 2 illustrates the sensitivity of estimated *f* values (mixing-model output) to uncertainties in end-member compositions. The mixing model is relatively insensitive to analytical errors, which are small (∼0.01 and 0.1‰ for *S* and *δ^18^O*, respectively) relative to other sources of uncertainty. The model is most sensitive to potential systematic error introduced by incorrect assumptions regarding the SW end-member.

### Aragonite Saturation States

In situ aragonite saturation states (Ω_a_) were calculated using the CO2calc carbon calculator application [43]. Input parameters were the TA–*p*H data pair, carbonic acid dissociation constants (*p*K_1_ and *p*K_2_) from Lueker et al. [44], HSO_4_^−^ dissociation constants from Dickson [45], and the borate dissociation constant of Lee et al. [46] and other measured parameters such as *T, S*. The solubility product (*K*_sp_) for aragonite was derived from Mucci [47]. Internal consistency was established by comparing Ω_a_ derived using the TA–*p*H pair with Ω_a_ derived using the*T*CO_2_–*p*H pair or *T*CO_2_–*p*CO_2_ pair. On the basis of these consistency test results, we estimate a precision of ±0.05 for Ω_a_.

**Figure 3:**
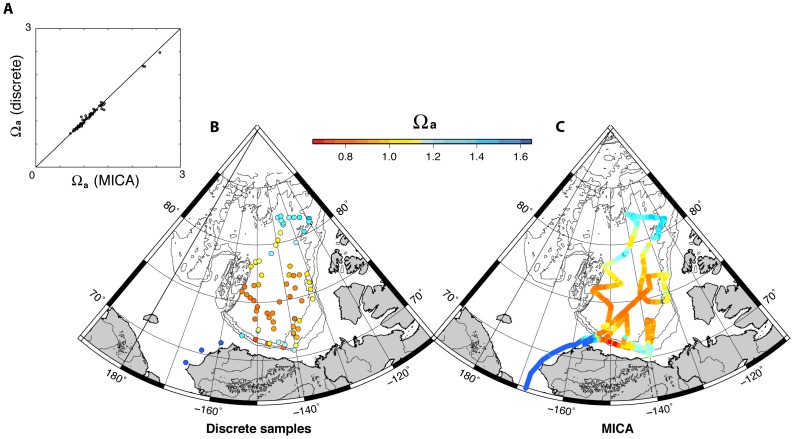
Aragonite saturation state, Ω_a_, calculated from discrete samples and from underway MICA readings. (A) Cross plot of discrete sample values with nearest-neighbor MICA values. (B) Map of 2010 discrete sample values. (C) Map of 2010 MICA values.

Values of Ω_a_ calculated from coincident MICA and discrete sample analyses (i.e., coincident *TA*–*p*H data pairs; *n* = 68) were statistically compared as an estimation of the equivalence of the two data sets. Spearman rank-order correlation shows the two data sets are highly and positively correlated (R^2^ = 0.985; *p*<0.001). The Mann-Whitney (α = 0.05) test for significant differences (*W* = 4712.0; *p* = 0.816) found no significant difference between the two data sets. Based on this equivalence (Figure 3A), we use Ω_a_ values calculated from the discrete samples (Figure 3B) to compare to other discrete sample measurements, and we use Ω_a_ values calculated from the MICA data (Figure 3C) to provide more detailed spatial resolution of Ω_a_ distributions.

The areal extent of aragonite undersaturation (Ω_a_<1) was estimated using ESRI ArcGIS software (version 10.1). Ω_a_ values calculated from the 2010 and 2011 cruises (>21,000 data points) were projected and then used to interpolate a raster map of aragonite undersaturation in the Makarov and Canada basins, both part of the Canadian Basin. A natural neighbor routine was used for the interpolation. The Makarov Basin (334,224 km^2^) and Canada Basin (1,524,664 km^2^) were delineated and measured by tracing the surrounding 1000 m isobath contour on the International Bathymetric Chart of the Arctic Ocean [48]. To determine the percent areal coverage of aragonite undersaturation, the interpolated area of undersaturation (371,525 km^2^) was divided by the area of the Canadian Basin (1,858,888 km^2^).

### Bacterial Production Rates

Bacterial production rates (*BP*; mg C m^−3^ d^−1^) were calculated using the following relationship:
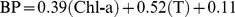
where *Chl-a* is chlorophyll *a* (µg L^−1^ d^−1^) and *T* is temperature (°C). This equation was derived from data previously collected in the Arctic Ocean during the same season as this study [49].

### Statistical Analyses

An initial assessment of the complete data set indicated that waters were not homogenous throughout the sampled area. The data set was therefore sorted into four regions based on differences in salinity and other measured parameters (Table 1): the Beaufort Sea, Makarov Basin, Sever Spur, and Canada Basin regions. The sample sites for the HLY1102 data set extend to higher latitudes than the HLY1002 data set and were therefore used to conservatively delineate these geographic boundaries. Comparisons for significant differences or similarities between each region were performed using the *f*_SIM_, *f*_MW_, *f*_SW_, Ω_a_, *p*CO_2_, *S, T, Chl-a,* and *BP* data sets. The data from each region were not normally distributed as determined by the Shapiro-Wilk normality test (α = 0.05). Accordingly, comparisons of variables were performed using the nonparametric Kruskal-Wallis one-way ANOVA (KW ANOVA, α = 0.05), followed by an all-pairwise multiple comparison of median values using Dunn’s method (α = 0.05). Dunn’s method for multiple comparisons determines which of the comparisons analyzed by KW ANOVA are significantly different. A *p*-value of <0.05 indicates a significant difference between the two median values. The Spearman rank-order method was used for all correlation calculations.

Rates of change of Ω_a_ were obtained by comparing September 1997 data [50] to the *Healy* 2010 and 2011 data. Surface *TA* and *T*CO_2_ from similar locations were used to calculate Ω_a_ as described above. Rate of change for a given location was then calculated as the slope of a linear fit of Ω_a_ vs. year. The 1997 *T*CO_2_ and *TA* data were analyzed on board using Dickson Certified Reference Material for every 20 samples to evaluate accuracy. Precision for the 1997 data was ∼0.1% for *T*CO_2_ and ∼0.2% for *TA*.

### Data Management

The data reported here are available at http://pubs.usgs.gov/ds/741/and http://pubs.usgs.gov/ds/748/.

## Results

### Spatial Coverage

In the course of the late-summer 2010 and 2011 *Healy* cruises, more than 34,000 water samples (flowthrough and discrete) were analyzed for *S*, *T*, *p*CO_2_, *Chl-a*, and *BP*, and calculated Ω_a_ (Figure 1). The resulting data sets are among the most comprehensive in the region (Previous work in the area shown in Figure 1 had collectively produced ∼19,900 data records). Sampling length intervals ranged from approximately 0.24 km to 10 km or more, depending on ship speed and sampling frequency. Table 2 shows median and range values for the HLY1102 data records that contain a complete suite of all of the following parameters: Ω_a_, *S*, *T*, *p*CO_2_, *f*_SIM_, *f*_MW_, *f*_SW_, *Chl-a*, and *BP*.

**Table 2:** Regional median values (and ranges) for 2011 surface water data and mixing-model output.

Parameter (units)	Beaufort Sea	Makarov Basin	Sever Spur	Canada Basin
	(*n* = 53)	(*n* = 49)	(*n* = 24)	(*n* = 103)
Ω_a_	1.15 (0.85–1.35)	1.52 (1.25–1.61)	1.25 (1.14–1.46)	1.04 (0.70–1.84)
*f_S_*_IM_	0.083 (–0.009–0.215)	−0.010 (–0.035–0.027)	0.041 (0.016–0.061)	0.080 (−0.024–0.219)
*f*_MW_	0.252 (0.170–0.307)	0.137 (0.116–0.119)	0.151 (0.132–0.177)	0.171 (0.054–0.256)
*f*_Sw_	0.664 (0.553–0.816)	0.877 (0.825–0.900)	0.805 (0.786–0.840)	0.742 (0.525–0.932)
*T* (°C)	5.91 (2.22–7.96)	–1.63 (−1.68– −1.49)	–1.50 (−1.58– −1.48)	−1.32 (−1.54−7.99)
*S*	23.51 (19.11–28.47)	30.61 (28.75–31.35)	28.28 (27.69–29.43)	26.25 (19.22–32.60)
*p*CO_2_ (µatm)	370.3 (310.6–386.1)	264.0 (242.7–330.4)	335.3 (331.9–338.5)	322.9 (247.4–554.9)
*p*H	8.05 (8.03–8.13)	8.19 (8.13–8.22)	8.15 (8.10–8.21)	8.07 (7.76–8.20)
*Chl-a* (µg·L^−1^)	0.19 (0.11–0.31)	0.29 (0.19–0.46)	0.22 (0.20–0.26)	0.17 (0.09–1.86)
*BP* (mg C·L^−1^·d^−1^)	3.27 (1.37–4.34)	0.62 (0.53–0.69)	0.59 (0.58–0.61)	0.53 (0.004–4.48)

### Regionally Distinct Seawater Properties

The four delineated regions of the western Arctic (Table 2) are the Beaufort Sea, Canada Basin, Makarov Basin, and Sever Spur regions. Although these names coincide with geographic regions rather than oceanographic regimes, the Beaufort Sea is predominantly shallow and coastal, while the other regions are predominantly deep basins. In pairwise comparisons, the regions exhibit significant differences in four or more of the seawater variables of Ω_a_, *S*, *T*, *p*CO_2_, *f*_SIM_, *f*_MW_, *f*_SW_, *Chl-a*, and *BP* (Table 3).

**Table 3:** Dunn’s Method *Q*-statistic results for pairwise multiple comparisons of the four Arctic Ocean regions.

Pairwise Comparison	Ω_a_	*f*_SIM_	*f*_MW_	*f_S_*_W_	*p*CO_2_	*T*	*S*	*Chl-a*	*BP*
Beaufort Sea vs. Makarov Basin	X	X	X	X	X	X	X	X	X
Beaufort Sea vs. Sever Spur	*ns*	X	X	X	X	X	X	*ns*	X
Beaufort Sea vs. Canada Basin	*ns*	*ns*	X	X	X	X	X	*ns*	X
Makarov Basin vs. Sever Spur	X	X	*ns*	X	X	*ns*	X	*ns*	*ns*
Makarov Basin vs. Canada Basin	X	X	X	X	X	X	X	X	X
Sever Spur vs. Canada Basin	X	X	*ns*	X	*ns*	X	X	X	*ns*

A significant difference (*p*<0.05) between paired regions is denoted by an X, and a non-significant difference is denoted by *ns*. All comparisons were conducted at α = 0.05.

**Figure 4:**
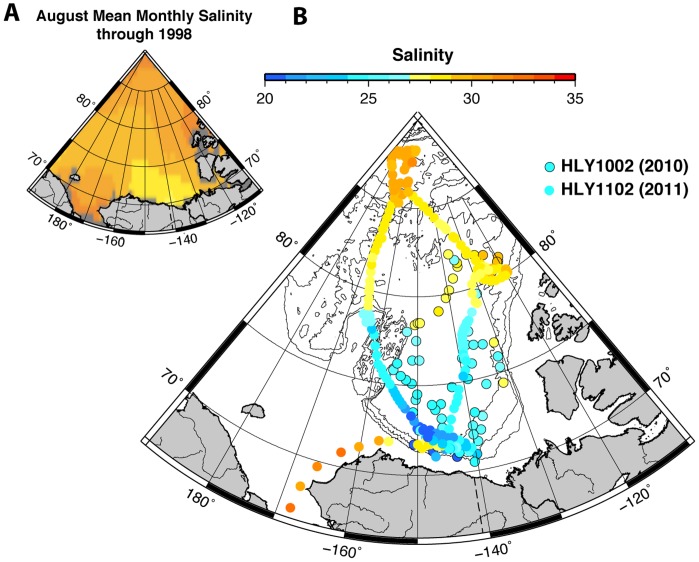
Surface salinity in the western Arctic Ocean. (A) Map showing August mean salinity (through 1997) at 10 m depth from the World Ocean Atlas 1998 [51]. Data provided by Physical Sciences Division, Earth System Research Laboratory, NOAA (http://www.esrl.noaa.gov/psd/). (B) Map of HLY1002 (2010) and HLY1102 (2011) discrete sample salinities.

**Figure 5:**
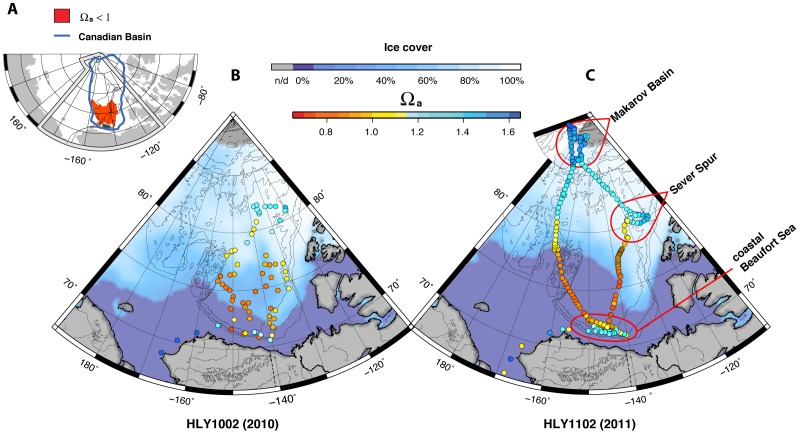
Surface water aragonite saturation state (Ω_a_) and ice cover in the western Arctic Ocean. (A) Canadian Basin (outlined in blue) and area of surface Ω_a_ undersaturation during 2010–2011 (red). (B) HLY1002 aragonite saturation state. (C) HLY1102 aragonite saturation state. Maps (B) and (C) also show average sea ice concentration (% ice cover) for August 2010 and September 2011, respectively [70]. The Makarov Basin, Sever Spur, and coastal Beaufort Sea regions are encircled in red; the remaining data points are assigned to the Canada Basin region. All Ω_a_ values are calculated from discrete sample data.

**Figure 6:**
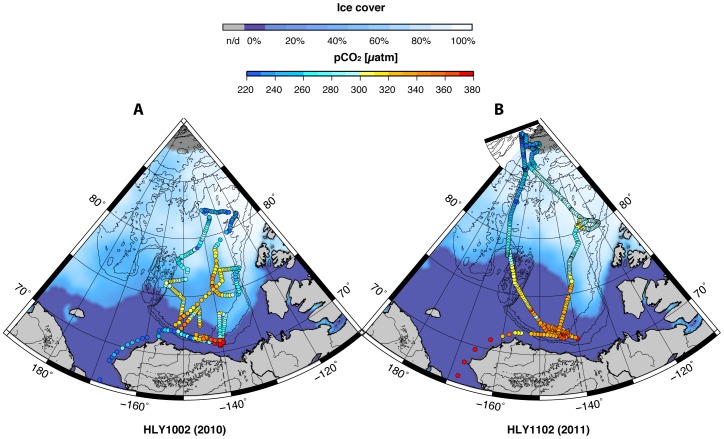
Surface *p*CO_2_ in the western Arctic Ocean. (A) HLY1002. (B) HLY1102. The data points show underway *p*CO_2_ values recorded at locations where discrete surface water samples were collected. The maps also show average sea ice concentration (% ice cover) for August 2010 and September 2011, respectively [70].

Figure 4 shows climatological August surface salinity through 1997 [52] and surface salinity encountered on the HLY1002 and HLY1102 cruises. The recent data show a generally lower *S* than is seen in the estimates of long-term means. The *Healy* data also show *S* increasing with increasing latitude (Figure 4) and ice cover (Figure 5). A concomitant increase in Ω_a_ with decreasing *p*CO_2_ occurred along this same latitudinal trend (Figures 5 and 6). An exception to this general pattern is the Beaufort Sea region, which shows higher Ω_a_ values in the samples near the coast.

### Aragonite Undersaturation and Water Mass Sources

Our calculations of Ω_a_ in the combined Canada and Makarov basins indicate that in the summers of 2010 and 2011, approximately 20% of the 1.9 million km^2^ surface area was undersaturated with respect to aragonite (i.e., Ω_a_<1; Figure 5). All observed undersaturation was confined to the Canada Basin and Beaufort Sea. Undersaturated water extended poleward from the Beaufort Sea to nearly 80°N between the Canadian Archipelago and the Chukchi Cap (over 3.7×10^5^ km^2^). Waters supersaturated with respect to aragonite were seen in the Makarov Basin and Sever Spur areas (1.14≤Ω_a_≤1.61) in association with nearly 100% sea ice cover and relatively low *p*CO_2_ (Figures 5 and 6). Pairwise statistical comparisons show that Ω_a_ values in these two regions were significantly greater than in the Canada Basin (Table 3). Beaufort Sea coastal waters had Ω_a_ values significantly lower than Makarov Basin waters.

**Figure 7:**
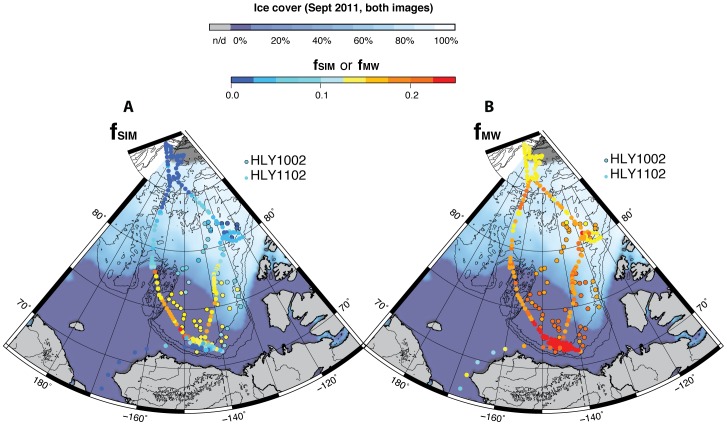
Modeled freshwater fractional contributions to surface waters of the western Arctic Ocean. (A) Fraction of sea ice melt, *f*_SIM_. (B) Fraction of meteoric water, *f*_MW_. The maps also show average sea ice concentration (% ice cover) during September 2011 [70].

Sea ice melt showed the greatest contribution to water masses in the Beaufort Sea and Canada Basin (*f*_SIM_ up to 0.219) and comparatively low contributions in the Makarov Basin and Sever Spur areas (*f*_SIM_ up to 0.061; Table 1, Figure 7). Values of *f*_MW_ were more homogenous throughout the study area but were generally lower in the Makarov Basin and Sever Spur areas (0.116–0.177) than in the Beaufort Sea and Canada Basin (0.054–0.307).

Surface water Ω_a_ was strongly and positively correlated with surface *S* in all localities (Table 4). Further, *S* in all localities was strongly but negatively correlated with the fraction of fresh water derived from sea ice meltwater (*f*_SIM_) and/or meteoric water (*f*_MW_; Table 4). Values of *f*_MW_ showed a consistent negative correlation to Ω_a_ throughout the entire western Arctic, including waters in ice-covered areas such as the Makarov Basin.

**Table 4:** Spearman rank-order correlations between the Arctic Ocean regions.

Region	Parameter	*f*_SIM_	*f*_MW_	*f*_SW_	T (°C)	*S*	*p*CO_2_	*Chl-a* (mg m^−3^)	*BP* (mg C m^−3^ d^−1^)
Beaufort Sea (BS)	Ω_a_	−0.908**	−0.370**	0.797**	*ns*	0.782**	*ns*	*ns*	*ns*
Makarov Basin (MB)		−0.370**	−0.791**	0.830**	−0.788**	0.849**	−0.827**	*ns*	0.563**
Sever Spur (SS)		*ns*	−0.843**	0.825**	−0.567**	0.849**	*ns*	*ns*	0.526**
Canada Basin (CB)		−0.866**	−0.517**	0.837**	0.349**	0.830**	*ns*	0.493**	0.504**
	BS	*f*_SIM_	0.534**	−0.932**	*ns*	−0.920**	*ns*	−0.391**	*ns*
	MB		*ns*	−0.667**	0.323*	−0.620**	*ns*	*ns*	−0.343*
	SS		*ns*	−0.477*	0.410*	−0.410*	*ns*	*ns*	*ns*
	CB		0.542**	−0.945**	−0.336**	−0.935**	*ns*	−0.467**	−0.517**
		BS	*f*_MW_	−0.804**	0.671**	−0.823**	*ns*	*ns*	0.670**
		MB		−0.759**	0.845**	−0.795**	0.562**	*ns*	−0.377*
		SS		−0.726**	0.540*	−0.775**	*ns*	*ns*	−0.517**
		CB		−0.786**	−0.500**	−0.804**	−0.453**	−0.531**	−0.582**
			BS	*f*_SW_	−0.365*	0.999**	*ns*	0.363*	−0.358*
			MB		−0.840**	0.996**	−0.433*	*ns*	0.504**
			SS		−0.783**	0.997**	*ns*	0.511*	0.687**
			CB		0.441**	0.999**	0.198*	0.549**	0.606**
				BS	*T*	−0.384**	0.706**	*ns*	*ND*
				MB		−0.869**	0.496**	*ns*	*ND*
				SS		−0.778**	*ns*	−0.675**	*ND*
				CB		0.448**	0.663**	0.779**	*ND*
					BS	*S*	ns	0.358*	−0.378*
					MB		−0.464*	*ns*	0.495**
					SS		*ns*	0.501*	0.687**
					CB		0.212*	0.553**	0.611**
						BS	*p*CO_2_	*ns*	0.709**
						MB		*ns*	−0.417*
						SS		*ns*	*ns*
						CB		0.389**	0.587**

A significant correlation between parameters is denoted by * at *p*<0.05 or by ** at *p*<0.001. An entry of *ns* indicates no significant correlation. An entry of *ND* indicates no data.

### Aragonite Undersaturation and Biological Processes

Aragonite saturation state can be influenced by not only air–sea CO_2_ exchange but also biological activities such as carbon fixation and respiration. Photoautotrophic organisms may remove CO_2_ from the water, thereby tending to increase Ω_a_. Respiration, in contrast, adds CO_2_, thereby tending to decrease Ω_a_. During our late summer cruises, *Chl-a*, which is indicative of photoautotrophic activity, showed no significant correlation with Ω_a_ except in the Canada Basin (Table 4), where the correlation was positive. *Chl-a* and *p*CO_2_ also positively covaried in the Canada Basin. The *p*CO_2_: Ω_a_ relationship was significantly and negatively correlated in the Makarov Basin and showed no significant association in the other regions.

## Discussion

Projections of the extent and rate of Arctic Ocean acidification and the associated ecosystem changes require models based on causal relationships and complex feedbacks among rapidly changing physical, chemical, and biological processes. Model formulation and validation both require extensive data sets. Our baseline study significantly expands the body of data available for such work and also provides the first high-resolution data from higher latitude waters (up to 88°48′ N into the Makarov Basin; Figure 1). Previous work has focused primarily on coastal areas of the southern Canada Basin and the Bering and Beaufort Seas [20], [22], [25], [51]–[55]. The new data provide a basis for detecting future change and analyzing trends in surface water chemistry.

Combined with older data, the 2010–2011 measurements can also provide insight into changes that have already occurred in the Arctic, primarily over the past two decades. (Canadian Basin carbonate chemistry data from before the 2000s are sparse.) For example, a comparison with 1994 cruise data [50] indicates that surface *S* in the northern Canada Basin and the Makarov Basin (i.e., above ∼80°N) decreased only slightly over the 17 intervening years, from 31.48 in 1994 (*n = *33) to 30.61 in 2011 (*n = *49). Over roughly the same time period, a large decrease in average *S* occurred in the Beaufort Sea–southern Canada Basin (∼72–75°N), from 28.49 (late September 1997, *n* = 19; [50]) to 23.51 (September 2010, *n* = 53).

This marked surface freshening was accompanied by a large decrease in average Ω_a_, from 1.31 to 1.14, which suggests a link between Ω_a_ and dilution from sea ice melt in these areas. A number of locations that were supersaturated with respect to aragonite in 1997 were undersaturated in 2010 and 2011. Based on the limited data available from sites in the southern Canada Basin, we estimate that summer surface Ω_a_ decreased at a rate of 2.1% yr^−1^ between 1997 and 2010–nearly an order of magnitude greater than the average rate observed for the Pacific Ocean (0.36% yr^−1^; [56]).

### Effects of Sea Ice Melt on Aragonite Saturation State

The observed decrease in aragonite mineral saturation state in the southern Canada Basin since 1997 has been accompanied by a decrease in late summer salinity (Figure 4). Increased freshwater in the Arctic Ocean is known to have diluted surface mixed layer *S* over the past decade [57]–[61]. This freshening may have been enhanced in the western Arctic, where recent spin-up of the wind-driven convergence of the Beaufort Gyre may have increased freshwater retention [57], [60]. The strong vertical density gradient of the Arctic Ocean also acts to maintain low *S* in the surface mixed layer (∼0–50 m depth) throughout the seasons [18]. The cold Arctic halocline at ∼50–400 m depth reduces upward mixing of CO_2_-rich deep waters to the surface mixed layer [18], thus playing a significant role in maintaining low surface *S* and Ω_a_.

**Figure 8:**
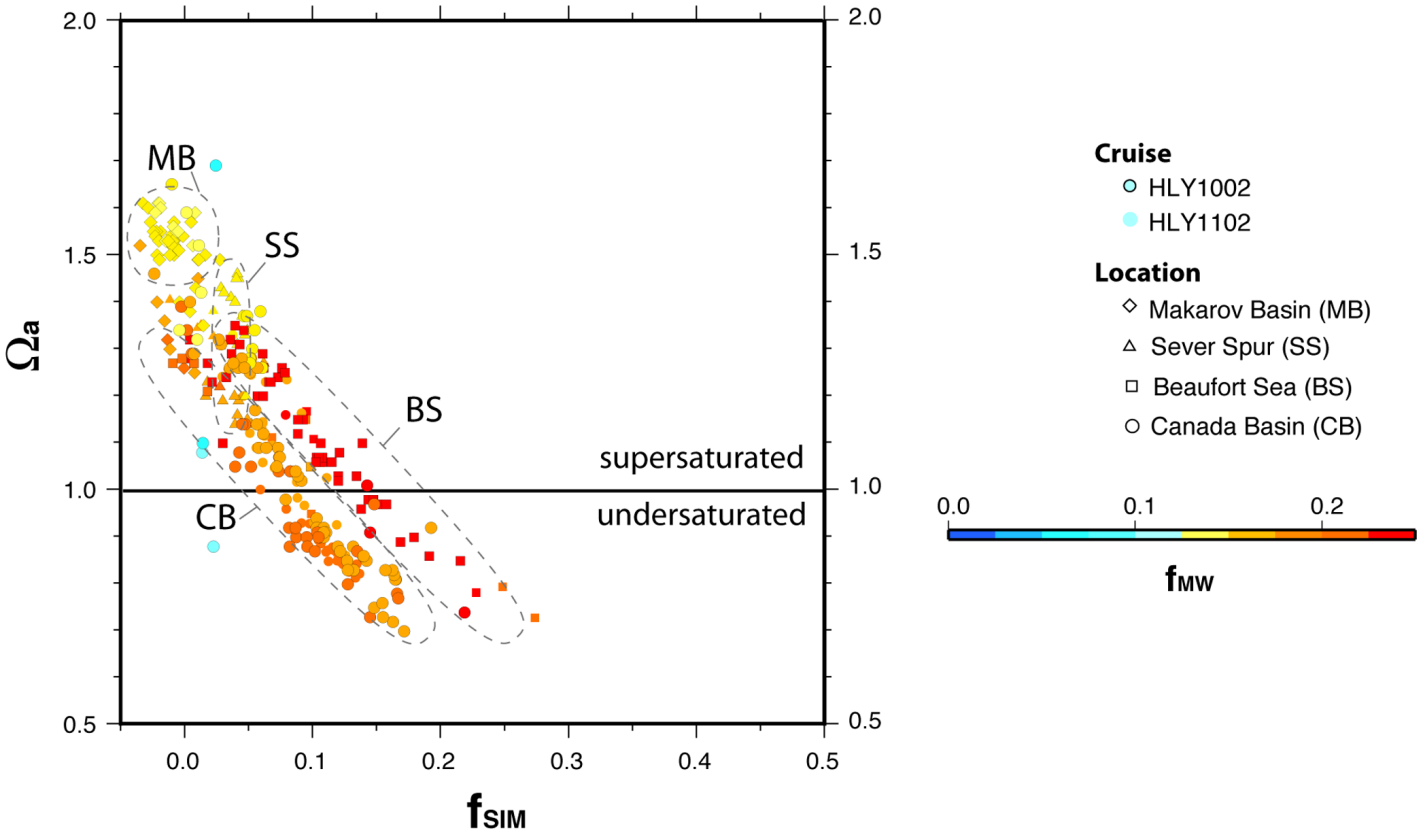
Aragonite saturation state and freshwater contributions in regions of the western Arctic Ocean. Comparison of aragonite saturation state (Ω_a_) to freshwater source fractions (*f*_SIM_ plotted on *x*-axis, *f*_MW_ coded to point color). Symbol shapes identify the basin, where the data were collected. Ellipses outline general data clusters from each region.

Comparisons of *S* and Ω_a_ show a significant correlation throughout the study area (Figures 4, 5, and 8; Table 4), suggesting that dilution with freshwater provides a direct mechanism for reducing saturation state [8], [21]. The water-mass mixing model distinguishes the proportion of freshwater derived from sea ice melt (*f*_SIM_) vs. meteoric water (*f*_MW_; i.e., terrestrial runoff and direct precipitation). Relatively high *f*_SIM_ was observed in areas south of ∼80°N (Figure 8A), notably in those areas where summer ice extent had recently decreased and particularly where multiyear ice had melted. The strong negative correlation between *f*_SIM_ and Ω_a_ in the Beaufort Sea and Canada Basin (Figure 8, Table 4) reflects the dominant influence predicted by dilution from sea ice meltwater on aragonite saturation state. Areas north of 80°N, such as the Makarov Basin and Sever Spur regions, were largely immune to the effects of sea ice melt on aragonite undersaturation, showing weak to no significant correlation between *f*_SIM_ and Ω_a_.

The influence of *f*_MW_ (Figure 7B) was more homogenous than *f*_SIM_ (Figure 7A). Within the relatively narrow *f*_MW_ range encountered, highest values occurred in Beaufort Sea coastal waters while lower values occurred in the ice-covered Makarov Basin. Overall, *f*_MW_ values were negatively correlated to Ω_a_ in all regions, which reflects the spatially consistent role of meteoric freshwater in reducing Ω_a_. Still, those correlations were relatively weak compared to the correlations between *f*_SIM_ and Ω_a_ in the Beaufort Sea and Canada Basin (Table 4), where Ω_a_ is strongly affected by sea ice meltwater.

The variable influence of freshwater sources across the regions is evident in the geographic distribution (clustering) of the data shown in Figure 8. For the data set as a whole, Ω_a_ is negatively correlated with *f*_SIM_, but regional distinctions are apparent. The Makarov Basin and Sever Spur areas are generally characterized by relatively supersaturated Ω_a_ values and generally low *f*_SIM_ (Tables 2, 4). The Canada Basin and Beaufort Sea data show a strong correlation between Ω_a_ and *f*_SIM_, but for a given *f*_SIM_ value, Beaufort Sea Ω_a_ values are higher than those from the Canada Basin. This offset is likely due to the fact that meteoric water from river runoff, which is more abundant in the coastal waters of the Beaufort Sea (Figure 7B), contributes higher concentrations of dissolved calcium, inorganic carbon, and carbonate alkalinity than does sea ice melt [22]. As a result, Beaufort Sea surface waters are more strongly supersaturated than Canada Basin waters for an equivalent contribution of freshwater from sea ice melt. This effect, observed strongly in the Beaufort Sea, is likely enhanced by the fact that North American rivers carry more calcium, inorganic carbon, and carbonate alkalinity than do Eurasian rivers [39]. Thus, the net effect of meteoric water on lowering Ω_a_ values is likely less significant in the southern Canada Basin and Beaufort Sea, where the Mackenzie River is the primary meteoric water source, than in the Makarov Basin (Figure 7B), where Eurasian rivers are the primary source. Over the past decade, the Makarov Basin has received increased input from these rivers [59]. Differences between pelagic and coastal waters may also be partially attributed to a pronounced upwelling event that occurred in the Beaufort Sea during late summer 2011 [52]. This coastal upwelling may account for a reduced MW role, resulting in somewhat higher regional Ω_a_ values in 2011 than in 2010.

Regarding the relative roles of *f*_MW_ and *f*_SIM_, it is worth noting that our sensitivity analysis (Figure 2) suggests that *f*_MW_ values may in some cases be overestimated due to the simplifying assumption that the SW end-member is Atlantic-derived. A portion of the freshwater attributed in our analysis to terrestrial runoff from Arctic rivers may in actuality derive from Bering Strait Inflow. However, the inference of a strong regional relationship between sea ice melt and low Ω_a_ remains robust, being relatively insensitive to model assumptions.

**Figure 9:**
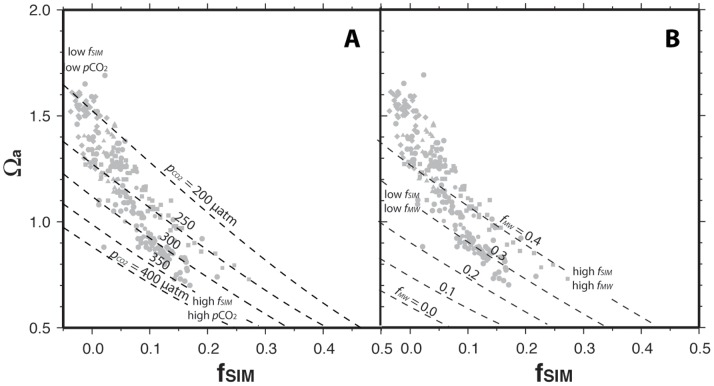
Modeled Ω_a_:*f*_SIM_ for conservative mixing of seawater and sea ice meltwater. (A) Effect of variable *p*CO_2_ with constant *f*_MW_ = 0.18. (B) Effect of variable *f*_MW_ with constant *p*CO_2_ = 372.6 µatm. The values of the constants are equal to the mid-range values observed during the 2011 cruise. The gray points show the data from Figure 8. For the freshwater end-member [33], [35], [39], [55], *TA*_MW_ = 1000 µmol kg^−1^, *S*_FW_ = 0, and *T*CO_2MW_ = 940 µmol kg^−1^. For the sea ice meltwater end-member [40], [71], *TA*_SIM_ = 540 µmol kg^−1^, *S*_SIM_ = 4, and *T*CO_2SIM_ = 300 µmol kg^−1^. Atlantic seawater is characterized according to mean values observed at the thermocline in the HLY1102 station data, 250–400 m depth: *TA*_SW_ = 2318.5 µmol kg^−1^, *S*_SW_ = 34.92, and *T*CO_2SW_ = 2156.6 µmol kg^−1^). All models are calculated at a seawater *T* of −1.47°C (median value of the HLY1102 data set).

The observed negative correlation between *f*_SIM_ and Ω_a_ (Figure 8) may convolve the two hypothesized effects of reduction of sea ice cover on Ω_a_–namely, dilution with meltwater and exposure to the CO_2_-rich atmosphere, both of which may act to reduce Ω_a_ in areas of recent sea ice melt. Figure 9A illustrates the combined roles of these two effects in a simple model of conservative mixing between end-member seawater and sea ice meltwater. Model lines show variation of the *f*_SIM_: Ω_a_ relationship under conditions in which *p*CO_2_ is varied while *f*_MW_ is held constant (dashed lines show model curves; gray points show observed data). The model lines show that for a given *f*_SIM_ contribution, a higher *p*CO_2_ corresponds to a lower saturation state. As predicted by the model, Ω_a_ is negatively correlated with *f*_SIM_, but the slope for the correlation of the observed data is steeper than model predictions. The greater slope associated with the observations is likely due to the combination of the two sea-ice–related effects on Ω_a_. For instance, waters represented by points at the left side of the data cluster derive predominantly from the Makarov Basin, where Ω_a_ is generally highest. These waters show low *f*_SIM_ values, due to minimal melting of sea ice, and also low *p*CO_2_ values, reflecting disequilibrium with the atmosphere (labeled “low *f*_SIM_, low *p*CO_2_” in Figure 9A). In contrast, waters in areas of recent sea ice melt, such as the Canada Basin or Beaufort Sea, show high *f*_SIM_ values and generally high *p*CO_2_ (labeled “high *f*_SIM_, high *p*CO_2_” in Figure 9A). The combination of the disequilibrium effect with the meltwater effect tends to produce a trend connecting the data from these two regions, with a steeper slope than is predicted by a model that includes seawater–meltwater mixing only.

Figure 9B shows a similar model of conservative mixing of seawater and sea ice meltwater, but in this case varying *f*_MW_ while holding *p*CO_2_ constant. Again, the model lines show that a higher fractional contribution of sea ice melt corresponds to a lower saturation state. However, the addition of meteoric water tends to partially counter the effect of SIM: for a given meltwater contribution (*f*_SIM_), increasing the meteoric contribution (*f*_MW_) corresponds to a higher saturation state. As with Figure 9A, the observed data show a steeper negative slope than that predicted by the simple two-component mixing model. However, *f*_SIM_ and *f*_MW_ are observed to be positively correlated throughout the Canada Basin and Beaufort Sea (Table 4). Connecting regions of “low *f*_SIM_, low *f*_MW_” and “high *f*_SIM_, high *f*_MW_” in Figure 9B suggests that the correlation between these variables would tend to counter the “steeper-than-predicted” slope of the correlation between *f*_SIM_ and Ω_a_ discussed above.

The model shown in Figure 9B also illustrates the proposed explanation for the positive offset of Ω_a_ values (for a given *f*_SIM_) from the Beaufort Sea as compared to the Canada Basin (Figure 8). Model calculations of the *f*_SIM_: Ω_a_ relationship are offset to higher Ω_a_ values as a result of high modeled *f*_MW_. This effect is seen predominantly in the Beaufort Sea. This effect is likely due to terrestrial contributions of dissolved calcium, inorganic carbon, and carbonate alkalinity from the Mackenzie River [39].

### Effects of Biological Activity on Aragonite Saturation State

Variation in Ω_a_ and *p*CO_2_ values show the effects of not only hydrodynamic forcing [24], regional sea ice melt, and terrestrial inputs of freshwater and CO_2_ [25], but also local biological processes. Consistent with previous Arctic Ocean surface water observations [62], our Makarov Basin measurements showed low CO_2_ partial pressures compared to atmospheric values. Seawater *p*CO_2_ ranged from 242 to 330 µatm; atmospheric values at the time were ∼380 µatm or higher. Seawater *p*CO_2_ values lower than atmospheric values have previously been attributed to (a) incomplete seawater equilibration with the present-day atmosphere and (b) cooling [8], [17], [20], but also (c) relatively low net community production in areas of offshore basins as compared to coastal waters [8].

Bacterial metabolism is the collective process of bacterial production (*BP*) (Table 2) and respiration (*BR*). Bacterial production pathways produce biomass, while respiration pathways generate energy for cellular processes. The *BR* reactions release CO_2_ when organic and inorganic C are substrates. The efficiency at which bacteria incorporate C into their biomass is a fraction of the total C utilized by the heterotrophic microbial community. This efficiency is described by the bacterial growth efficiency [*BGE* = *BP*/(*BP*+*BR*)] [63]. In general, BGE values range from 0.03 to 0.27 in open marine waters and from 0.09 to 0.45 in coastal waters. *BGE* values in the western Arctic Ocean have been shown to be relatively low, averaging only 0.07±0.09 [49], [64]. The low BGE indicates that a significant proportion of the utilized C is being used in respiration-related processes, which produce and release CO_2_.

Differences in the *BGE* of heterotrophic microbial communities in the four regions may explain the inconsistent correlations between *p*CO_2_ and *BP.* These quantities are positively correlated in the Beaufort Sea and Canada Basin regions but are negatively correlated in the Makarov Basin and Sever Spur regions (Table 4). A confounding influence on these inconsistent correlations may be the overwhelming contribution of atmospheric *p*CO_2_ exchange relative to the heterotrophic microbial community production of *p*CO_2_. The influence of heterotrophic microbial community respiration on *p*CO_2_ and thereby Ω_a_ would be detected only if the effects (i.e., increasing *p*CO_2_ and decreasing Ω_a_) were greater than the net effect of CO_2_ influences from the atmosphere, photoautotrophic communities, and freshwater additions.

### Seasonal Trends

Seasonal trends of Ω_a_ in the Arctic Ocean are generally poorly documented (for an exception see [65]). Winter data collected in 2011 and 2012 between ∼83°N and 87°N [62] provide, in combination with our Makarov Basin data, a rare opportunity for a seasonal comparison. In general, the winter and summer values of surface water Ω_a_ and *p*CO_2_ were comparable. In addition, Makarov Basin under-ice mixed layer water (15–40 m below the ice) was sampled in April 2012 along a transect of the Switchyard Program (data archived by the Advanced Cooperative Arctic Data Information Service, aoncadis.org). The under-ice winter water was characterized by *T* = −1.69±0.04°C, *S* = 31.0±0.7, *p*CO_2_ = 312±13 µatm, and Ω_a = _1.31±0.06. These data are consistent with our Makarov Basin summer data (Table 2), indicating little seasonal variation in the surface water chemistry of the Makarov Basin.

### Implications for Modeling of Future Chemical Changes

Models have projected that large areas of the Arctic Ocean will become undersaturated with respect to aragonite in the next decade [3]–[6]. The data presented here indicate that the area of undersaturation presently extends to approximately 20% of the Canadian Basin in the late summer months, when sea ice is near its minimum extent. A number of other studies have shown areas of aragonite undersaturation in the Canadian Archipelago and on the Beaufort Sea shelf [21], [23], [24].

Projection of future freshening and undersaturation in areas currently under perennial ice cover, such as the Makarov Basin, may be facilitated by water source identification based on the use of salinity and oxygen isotopic composition as conservative tracers (e.g., Figures 7–9). Our analysis provides insight into how sea ice meltwater and meteoric water influence aragonite saturation states. The negative correlation between Ω_a_ and *f*_SIM_ (Figure 8, Table 4) characterizes the dependence of saturation state on the melting of multiyear sea ice and suggests a pattern along a transect from the Beaufort Sea to the Makarov Basin. The Makarov Basin represents an end-member whose chemical composition is essentially free of the effects of reduced sea ice cover and input of additional freshwater from melting of multiyear ice, while the southern Canada Basin represents conditions resulting from reduced sea ice. Given that summer ice-free conditions are projected to expand in the near term [10]–[12], these spatial trends allow prediction of the conditions under which the carbonate chemistry of the Makarov Basin will resemble that of the southern Canada Basin. Predictive models of ocean acidification must account for not only the effects of equilibration with atmospheric CO_2_ but also freshwater dilution and biogeochemical processes.

Data from this study also suggest that an assumption of metabolic coupling or co-dependence between heterotrophic and photoautotrophic organisms in the Arctic Ocean is not appropriate for inclusion in predictive models of ocean acidification processes. Recent studies of the relation between bacterial production and primary production in coastal and pelagic Arctic waters have shown that these carbon cycling processes can proceed uncoupled [49], [66], [67]. These studies found that the degree of correlation depends on time of year, percentage of ice cover, hydrodynamic regime, bacterial abundance, and bacterial growth efficiency. The absence of predictable correlations between biological variables and Ω_a_ in this study indicate that complex interactions are driving the biogeochemical processes. Uncoupling of these processes and variability in their rates and efficiencies have important implications for not only the retention of C at shallower productive depths [68] but also for the formulation of models that predict the influence of heterotrophic and photoautotrophic processes on *p*CO_2_.

In conclusion, the data presented here collectively suggest that recent decreases in western Arctic Ocean Ω_a_ can be predominantly attributed to recent melting of multiyear sea ice and the associated seawater freshening and uptake of atmospheric CO_2_; biogeochemical processes exert an additional influence. Despite increasing awareness of rapid global change in the Arctic, much remains to be done with respect to predicting changes in marine surface water chemistry. For example, few data are available for the polar winter, and it is not known whether aragonite-undersaturated areas decrease in size with the seasonal freezing of sea ice. Also, while the effects of sea ice melt may diminish over the long term, the effects of increasing terrestrial runoff and direct precipitation are likely to persist [69]. Quantitative documentation of these processes in the Arctic Ocean is needed for refinement of the next generation of global ocean acidification models.
